# Multiple viral introductions: molecular characterization of influenza B virus in Wenzhou, Zhejiang, China, from 2011 to 2014 based on hemagglutinin and neuraminidase genes

**DOI:** 10.1007/s00705-015-2721-7

**Published:** 2016-01-02

**Authors:** Dong Chen, Xiaohong Wen, Yi Sun, Haiyan Mao, Yanjun Zhang, Yin Chen, Xinying Wang, Baochang Sun, Xin Wang, Xiaoming Zhang

**Affiliations:** Wenzhou Center for Disease Control and Prevention, Wenzhou, Zhejiang China; First People’s Hospital of Huzhou, Huzhou Teachers College, Huzhou, Zhejiang China; Zhejiang Provincial Center for Disease Control and Prevention, 3399 Binsheng Road, Hangzhou, 310051 Zhejiang China

**Keywords:** Influenza B virus, Molecular epidemiology, Phylogenetic, Victoria, Yamagata

## Abstract

Influenza B virus is a major causative agent of respiratory disease in humans. Our study of an outbreak of influenza B virus in Wenzhou from 2011 to 2014 revealed that 163 (5.58 %) of 2921 samples were influenza B positive. Sequencing of the hemagglutinin and neuraminidase genes showed substitutions at the amino acid level. Phylogenetic analysis revealed co-circulation of the B/Victoria and B/Yamagata lineages in the Wenzhou area from 2011 to 2014. Multiple viral introductions from both Chinese and international sources played important roles in endemic co-circulation and transmission in coastal southeastern China.

**Electronic supplementary material:**

The online version of this article (doi:10.1007/s00705-015-2721-7) contains supplementary material, which is available to authorized users.

## Introduction

Influenza B virus has been a major pathogen in seasonal influenza outbreaks and has caused respiratory infections in humans globally. It was first isolated in 1940 during an epidemic in the USA (B/Lee/40). Influenza B virus is a member of the family *Orthomyxoviridae* and is closely related to influenza A viruses, which are similar in viral structure, genome organization and epidemiology [1–4]. Influenza B virus differs from influenza A virus, which has a diversity of subtypes according to surface glycoproteins, in having no subtypes, but it has been separated into two main antigenically distinct lineages, Victoria (B/Victoria/2/87-like) and Yamagata (B/Yamagata/16/88-like), since 1983, based on an analysis of the hemagglutinin gene [5]. Many studies have reported both types to have been predominant during different periods and in different geographic regions worldwide [2, 6, 7].

Wenzhou, a city in southeastern Zhejiang Province, China, includes four districts and 10 counties and is one of the important economic and business centers in Zhejiang. Infectious diseases such as pandemic H1N1 and foot-and-mouth disease have been monitored in Wenzhou, and several outbreaks of these pathogen-caused illnesses were dealt with during the last decade according to surveillance systems established by public health departments in China. Influenza B has now become one of the major public-health problems, as there have been many sporadic cases in recent years. Mutations in both the hemagglutinin (HA) and neuraminidase (NA) genes have allowed influenza B virus to circumvent the immune response in humans, to persist in human populations, to circulate in an endemic environment, and to cause recurrent seasonal epidemics [8–11]. Therefore, by combining the results of molecular and phylogenetic data, we attempted to determine (1) the molecular characteristics of both the hemagglutinin and neuraminidase genes and (2) the phylogenetic pattern of the influenza B virus in the Wenzhou area.

## Material and methods

This study was approved by the ethics committee of the Zhejiang Provincial Center for Disease Control and Prevention (ZJCDC), China. Following the ‘Surveillance Program of Influenza in China’, published by the National Health and Family Planning Commission (NHFPC), throat swabs and/or nasopharyngeal samples were collected in local hospitals and delivered to the ZJCDC from 2011 to 2014. In total, 2921 samples were obtained from patients exhibiting flu-like symptoms.

Viral RNA was extracted using an RNeasy Mini Kit (Roche) according to the manufacturer’s instructions. Influenza B virus infection was identified and genotyped by multiplex real-time PCR reactions using an AgPath-IDTM One-Step RT-PCR Kit (Life Technologies) following the protocol for the surveillance program.

Positive specimens were cultured in Madin-Darby canine kidney (MDCK) cells, a gift from the National CDC, for 5 to 7 days. Specific-pathogen-free embryonated chicken eggs were also used for virus isolation. Six 9- to 11-day-old chicken embryos were each inoculated with 300 µl of sample by the chorioallantoic sac route. The eggs were incubated for 48 hours at 35 °C. Cultured supernatants and allantoic fluids were tested by hemagglutination inhibition (HAI). Samples testing negative for hemagglutination were processed a second time.

Positive samples were subjected to RT-PCR amplification and sequencing of the hemagglutinin and neuraminidase genes. RT-PCR reactions for both the hemagglutinin (HA) and neuraminidase (NA) genes were done according to the surveillance program of Takara’s kit (Table S1). Sequencing was performed using an ABI 3730xl DNA Analyzer. All virus sequences have been deposited in the Global Initiative on Sharing All Influenza Data (GISAID) database (EPI630146-EPI630185).

Both the HA and NA gene were assembled and aligned along with additional sequences downloaded from GenBank. Variant positions in the nucleotide and amino acid sequences were checked using Geneious 4.8.5 (http://www.geneious.com). Identical indexes for both HA and NA were calculated using DNAStar Lasergene v7.1 (http://www.dnastar.com). Dataset-specific models that were selected using the Akaike information criterion in Modeltest 3.7 were analyzed [12]. Maximum-likelihood (ML) analysis was done using RAxML v7.2.8 (http://sco.h-its.org/exelixis/software.html). The optimal ML tree and bootstrap percentages (BP) were estimated in the same run. The ML BP values were obtained from 1000 bootstrap replicates using the rapid bootstrap algorithm. BEAST 1.6 (http://beast.bio.ed.ac.uk/Main_Page) was employed to date the divergence of the tree branches in each phylogenetic tree (HA and NA) based on an uncorrelated exponential distributed relaxed-clock model for our sample [13]. A burn-in of 10 % was used, and the convergence of all parameters was assessed using the software TRACER within the BEAST package.

## Results and discussion

We collected and tested 2921 samples, including throat swabs and nasopharyngeal swabs from across the Wenzhou area of Zhejiang Province from 2011 to 2014. One hundred sixty-three were positive for influenza B virus, a rate of 5.58 % (163/2921) for all samples (Table S2). This rate was higher than those observed in Thailand and the United States during a similar period of time [7, 14]. Most influenza B virus infections occurred in 2012, at a rate of 8.85 %, while the fewest cases were in 2014 (4.09 %). The majority of samples confirmed influenza B virus infections among children under 10 years of age at a rate of 47.23 % (77/163), which is in line with previous reports [15].

We detected many amino acid substitutions in both the HA and NA segment matrices, since variations in these sequences play a major role in pathogenicity of viral strains (Table 1). Sequence variations in the HA protein were divided into Yamagata and Victoria groups. Twenty-seven substitutions in the HA segment in the Yamagata lineage were detected, while in the Victoria lineage, there were 15 substitutions. A previous study reported four major antigenic epitopes – 120-loop (116-137), 150-loop (141-150), 160-loop (162-167) and 190-helix (194-202) – on the membrane distal domain of the HA1 region of the influenza B virus [11]. We observed many mutations in these important areas: for the Yamagata lineage, we found P123A, N131K and H137Q in the 120-loop area, N141D and R144K in the 150-loop area, R164K and N165S/I in the 160-loop area, and both T196A and K197E in the 190-helix area. We detected only T144N in the 150-loop in the Victoria lineage. A previous study indicated that the 120-loop (116-137) epitope dictates the antigenicity of HA [16]. We found that most mutations were in the 120-loop, especially in the Yamagata lineage, which was in line with several previous studies [11, 17]. N131Y detected in this study was considered a potential alteration in antigenicity of the Wenzhou strains. Variants in the 150-loop, 160-loop and 190-helix also obtained here, may contribute to differences in avoiding antigen recognition by neutralizing antibodies and to maintaining the structural integrity of some key proteins, such as receptor-binding proteins [7, 18]. Forty-two mutations were detected in the NA segment matrix constituted by the Wenzhou sequences when compared to both B/Yamagata/16/1988 and B/Victoria/2/1987. Substitutions found at positions 116, 117 and 402 were considered to be related to sensitivity to NA inhibitors and the function of drug resistance [4, 7]. No biological function has been suggested for many other amino acid changes identified in the NA segment. Pairwise identities were calculated according to different matrices in different segments. In the Victoria lineage, 97.5 % pairwise identity was determined, while 97.7 % was determined in the Yamagata lineage. Pairwise identity was estimated as 93.4 % in the total HA segment and 95.8 % in the NA segment.

**Table 1:** Substitutions detected at the amino acid level in the HA and NA segments. A, HA segment in the Yamagata lineage; B, HA segment in the Victoria lineage; C, NA segment. All sequences were compared to reference sequence listed in the first row. A dot (.) denotes same amino acid at the same position in the reference sequence

A. Yamagata													
							1	1	1	1	1	1	1
	5	6	7	8	8	9	2	3	3	4	4	6	6
	5	3	1	3	8	1	3	1	7	1	4	1	4
B/Wenzhou/1205/2013	Y	K	D	G	V	T	A	N	Q	D	K	A	K
B/Wenzhou/1823/2013	.	R	.	.	.	.	P	K	.	.	.	.	.
B/Wenzhou/187/2012	.	R	.	.	.	.	P	K	.	.	.	.	.
B/Wenzhou/1206/2013	.	.	.	.	.	.	.	.	.	.	.	.	.
B/Wenzhou/1194/2014	.	R	.	.	.	.	P	K	.	.	.	.	.
B/Wenzhou/196/2014	.	R	.	.	.	.	P	K	.	.	.	.	.
B/Wenzhou/1300/2013	.	.	.	.	.	.	.	.	.	.	.	.	.
B/Wenzhou/1784/2013	.	.	.	.	.	.	.	.	.	.	.	.	.
B/Wenzhou/1801/2013	.	R	.	.	.	.	P	K	.	.	.	.	.
B/Wenzhou/1790/2013	.	R	.	.	.	.	P	K	.	.	.	.	.
B/Wenzhou/13901/2013	.	R	.	.	.	.	P	K	.	.	.	.	.
B/Wenzhou/13902/2013	.	.	.	.	.	.	.	.	.	.	.	.	.
B/Yamagata/16/1988	H	.	N	A	M	I	P	.	H	N	R	V	R

A. Yamagata														
	1	1	1	1	1	1	2	2	2	2	2	3	3	3
	6	7	8	8	9	9	1	1	4	4	6	1	1	2
	5	7	0	1	6	7	1	7	4	7	6	2	3	7
B/Wenzhou/1205/2013	S	K	N	K	A	E	N	N	G	D	M	E	K	E
B/Wenzhou/1823/2013	I	.	Y	.	T	.	.	S	D	.	.	.	E	K
B/Wenzhou/187/2012	I	.	Y	.	T	.	.	S	.	.	.	.	E	K
B/Wenzhou/1206/2013	.	.	.	.	.	.	.	.	.	.	.	.	.	.
B/Wenzhou/1194/2014	I	.	Y	.	T	.	.	S	D	.	.	.	E	K
B/Wenzhou/196/2014	I	.	Y	.	T	.	.	S	D	.	.	.	E	K
B/Wenzhou/1300/2013	.	.	.	.	.	.	.	.	.	.	.	.	.	.
B/Wenzhou/1784/2013	.	.	.	.	.	.	.	.	.	.	.	.	.	.
B/Wenzhou/1801/2013	I	.	Y	.	T	.	.	S	D	.	.	.	E	K
B/Wenzhou/1790/2013	I	.	Y	.	T	.	.	S	D	.	.	.	E	K
B/Wenzhou/13901/2013	I	.	Y	.	T	.	.	S	D	.	.	.	E	K
B/Wenzhou/13902/2013	.	.	.	.	.	.	.	.	.	.	.	.	.	.
B/Yamagata/16/1988	N	R	K	T	T	K	D	K	D	N	V	A	.	.

B. Victoria															
							1	1	1	1	1	1	2	2	2
	6	8	8	9	9	9	0	4	5	8	8	9	1	1	5
	3	3	8	0	5	6	3	4	2	0	7	0	2	7	0
B/Wenzhou/820/2006	E	G	T	N	R	V	R	N	I	N	S	I	N	A	T
B/Wenzhou/1107/2012	.	.	.	K	.	.	.	.	.	K	P	.	.	.	.
B/Wenzhou/166/2005	.	.	.	.	.	.	.	.	.	.	.	.	.	.	.
B/Wenzhou/713/2006	.	.	.	.	.	.	.	.	.	.	.	.	.	.	.
B/Wenzhou/821/2006	.	.	.	.	.	.	.	.	.	.	.	.	.	.	.
B/Wenzhou/1467/2011	.	.	.	K	.	.	.	.	.	K	P	.	.	.	.
B/Wenzhou/1455/2011	.	.	.	K	.	.	.	.	.	K	P	.	.	.	.
B/Wenzhou/1448/2011	.	.	.	K	.	.	.	.	.	K	P	.	.	.	.
B/Wenzhou/15/2012	.	.	.	K	.	.	.	.	.	K	P	.	.	.	.
B/Victoria/2/1987	K	A	M	T	K	A	K	T	V	.	P	V	S	V	A

C. NA																					
																	1	1	1	1	1
	3	4	5	5	5	5	5	6	6	7	7	8	8	8	8	9	1	1	5	7	9
	7	4	1	2	4	6	9	0	1	3	5	0	1	3	6	8	6	7	8	0	6
B/Wenzhou/1194/2014	S	L	P	S	E	T	T	M	P	V	R	G	V	L	P	P	T	K	G	K	K
B/Wenzhou/1467/2011	.	S	S	P	.	.	.	.	.	.	.	.	.	.	.	.	.	.	.	.	.
B/Wenzhou/196/2014	.	.	.	.	.	.	.	.	.	.	.	.	.	.	.	.	.	.	.	.	.
B/Wenzhou/1206/2013	.	S	S	Q	.	.	.	.	.	.	.	.	.	.	.	.	.	.	E	.	.
B/Wenzhou/1448/2011	.	S	S	P	.	.	.	.	.	.	.	.	.	.	.	.	.	.	.	.	.
B/Wenzhou/1455/2011	.	S	S	P	.	.	.	.	.	.	.	.	.	.	.	.	.	.	.	.	.
B/Wenzhou/15/2012	.	S	S	P	.	.	.	.	.	.	.	.	.	.	.	.	.	.	.	.	.
B/Wenzhou/187/2012	.	S	S	R	.	.	.	.	.	.	.	.	.	.	.	.	.	.	E	.	R
B/Wenzhou/166/2005	.	S	.	.	.	.	.	.	.	.	.	.	.	.	.	.	.	.	.	.	.
B/Wenzhou/226/2006	.	S	.	.	.	.	.	.	.	.	.	.	.	.	.	.	.	.	.	.	.
B/Wenzhou/713/2006	.	S	.	.	.	.	.	.	.	.	.	.	.	.	.	.	.	.	.	.	.
B/Wenzhou/820/2006	.	S	.	.	.	.	.	.	.	.	.	.	.	.	.	.	.	.	.	.	.
B/Wenzhou/1107/2012	.	.	.	.	.	.	.	.	.	.	.	.	.	.	.	.	.	.	.	.	.
B/Wenzhou/1801/2013	.	.	.	.	.	.	.	.	.	.	.	.	.	.	.	.	.	.	.	.	.
B/Wenzhou/1823/2013	.	.	.	.	.	.	.	.	.	.	.	.	.	.	.	.	.	.	.	.	.
B/Wenzhou/1784/2013	.	S	S	Q	.	.	.	.	.	.	.	.	.	.	.	.	.	.	E	.	.
B/Wenzhou/1790/2013	.	.	.	.	.	.	.	.	.	.	.	.	.	.	.	.	.	.	.	.	.
B/Wenzhou/13901/2013	.	.	.	.	.	.	.	.	.	.	.	.	.	.	.	.	.	.	.	.	.
B/Wenzhou/13902/2013	.	S	S	Q	.	.	.	.	.	.	.	.	.	.	.	.	.	.	E	.	.
B/Yamagata/16/1988	.	S	S	P	.	.	K	V	.	.	.	.	M	.	S	Q	.	R	E	.	R
B/Victoria/2/1987	L	S	S	P	K	I	.	.	S	A	H	E	M	F	.	Q	A	.	E	N	R

C. NA																					
	2	2	2	2	2	2	2	2	3	3	3	3	3	3	3	3	4	4	4	4	4
	0	1	3	5	6	7	8	8	3	3	5	5	8	8	9	9	0	0	0	1	4
	8	2	5	8	0	2	1	2	0	9	2	5	5	8	4	9	2	5	6	4	6
B/Wenzhou/1194/2014	N	L	N	V	E	M	T	Q	K	D	G	S	K	G	N	A	D	A	F	E	E
B/Wenzhou/1467/2011	.	.	.	.	.	I	V	K	E	N	D	.	M	.	D	.	.	T	.	K	.
B/Wenzhou/196/2014	.	.	.	.	.	.	.	.	.	.	.	.	.	.	.	.	.	T	.	.	.
B/Wenzhou/1206/2013	S	.	D	.	.	I	V	K	E	N	D	.	M	.	D	T	E	.	L	.	K
B/Wenzhou/1448/2011	.	.	.	.	.	I	V	K	E	N	D	.	M	.	D	.	.	T	.	K	.
B/Wenzhou/1455/2011	.	.	.	.	.	I	V	K	E	N	D	.	M	.	D	.	.	T	.	K	.
B/Wenzhou/15/2012	.	.	.	.	.	I	V	K	E	N	D	.	M	.	D	.	.	T	.	K	.
B/Wenzhou/187/2012	S	.	D	.	.	I	V	K	E	N	D	.	M	.	D	T	E	.	L	.	K
B/Wenzhou/166/2005	.	.	.	.	.	I	I	K	E	.	.	.	M	.	D	.	.	.	.	.	.
B/Wenzhou/226/2006	.	.	.	.	.	I	I	K	E	.	D	.	M	.	D	.	.	T	.	.	.
B/Wenzhou/713/2006	.	.	.	.	.	I	I	K	E	.	.	.	.	.	D	.	.	V	.	.	.
B/Wenzhou/820/2006	.	.	.	.	.	I	I	K	E	.	.	.	.	.	D	.	.	.	.	.	.
B/Wenzhou/1107/2012	.	.	.	.	.	.	.	.	.	.	.	.	.	.	.	.	.	.	.	.	.
B/Wenzhou/1801/2013	.	.	.	.	.	.	.	.	.	.	.	.	.	.	.	.	.	T	.	.	.
B/Wenzhou/1823/2013	.	.	.	.	.	.	.	.	.	.	.	.	.	.	.	.	.	T	.	.	.
B/Wenzhou/1784/2013	S	.	D	.	.	I	V	K	E	N	D	.	M	.	D	T	E	.	L	.	K
B/Wenzhou/1790/2013	.	.	.	.	.	.	.	.	.	.	.	.	.	.	.	.	.	T	.	.	.
B/Wenzhou/13901/2013	.	.	.	.	.	.	.	.	.	.	.	.	.	.	.	.	.	.	.	.	.
B/Wenzhou/13902/2013	S	.	D	.	.	I	V	K	E	N	D	.	M	.	D	T	E	.	L	.	K
B/Yamagata/16/1988	S	I	D	I	K	I	V	E	E	.	D	R	M	E	D	T	.	.	P	K	K
B/Victoria/2/1987	S	I	D	I	K	I	V	E	E	.	E	R	M	E	D	T	.	.	P	K	K

**Fig. 1:**
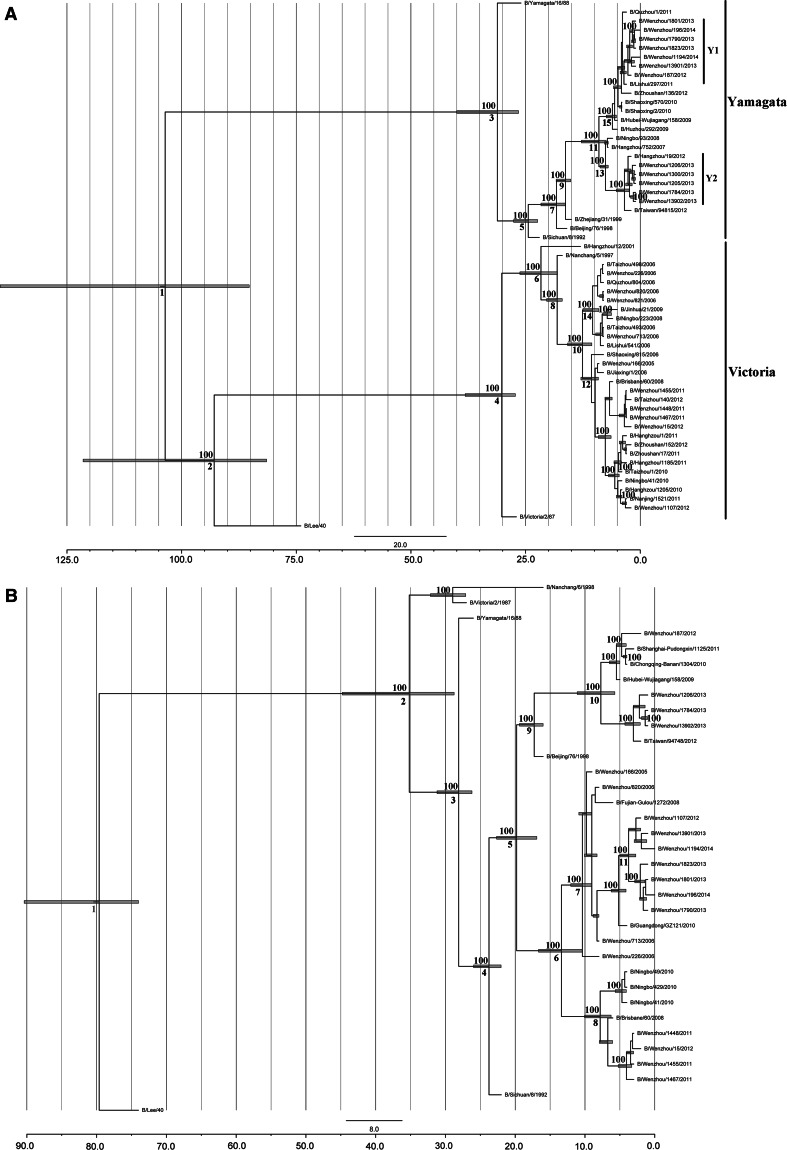
Maximum-likelihood tree for the HA and NA segments of influenza B virus. Bootstrap values based on 1000 replicates are indicated above the branches. Blue numbers below the branches show the time of the most recent common ancestor for each node. Blue bars on nodes are **95** **% highest posterior density (HPD) intervals**. A, HA segment; B, NA segment (color figure online)

Phylogenetic analysis of HA showed that influenza B viruses in the Wenzhou area from 2011 to 2014 separated into two main lineages, Yamagata and Victoria, according to the hemagglutinin gene. In the Yamagata lineage, most sequences, including the Wenzhou-area samples, were clustered into one monophyletic clade with the most common recent ancient time in the year 2004 (Fig. 1 and Table 2). Within this clade, sequences from the Wenzhou area formed two monophyletic clades: Y1 and Y2. Strains from Lishui (Lishui/297/2011) and Hangzhou (Hangzhou/19/2012) showed high homology to Y1 and Y2, respectively. The phylogenetic pattern for the Wenzhou sequences from 2011 to 2014 in the Victoria lineage showed different topological structures, as strains from Wenzhou scattered into different clades in the Victoria part. Strains mainly from 2011 to 2012 formed a monophyletic clade with sister strains from other locations such as Taizhou, Nanjing and Brisbane. The NA phylogenetic tree showed that sequences from the Wenzhou area from 2011 to 2014 clustered into one large monophyletic clade with other NA sequences from outside Wenzhou, such as from Shanghai, Beijing, Chongqing, Hubei, Taiwan, Fujian, Guangdong and Brisbane, with an estimated emergence time of 1994.

**Table 2:** Time of the most recent common ancestors for each node on phylogenetic trees of both HA and NA segments with their 95 % HPD value

Node position	Tmrca with 95 % HPD value	Estimated year with 95 % HPD value
HA		
1	100.423 (80.4821-130.252)	1914 (1884-1934)
2	89.99 (78.4315-106.5276)	1924 (1907-1936)
3	31.969 (26.4292-41.1348)	1982 (1973-1988)
4	31.343 (27.1216-38.9477)	1983 (1975-1987)
5	24.719 (22.1872-28.1818)	1989 (1986-1992)
6	21.53 (17.9808-25.8485)	1993 (1988-1996)
7	18.539 (16.2876-21.5348)	1995 (1993-1998)
8	18.317 (17.0103-20.4987)	1996 (1994-1997)
9	16.454 (15.1393-18.2565)	1998 (1996-1999)
10	12.8 (10.1194-15.6577)	2001 (1998-2004)
11	9.705 (7.5551-13.0773)	2004 (2001-2006)
12	7.639 (6.366-9.0507)	2006 (2005-2008)
13	7.738 (7.0003-8.7973)	2006 (2005-2007)
14	10.389 (8.9321-12.1783)	2004 (2002-2005)
15	6.246 (5.2909-7.4976)	2008 (2007-2009)
NA		
1	80.988 (74.0005-96.7082)	1933 (1917-1940)
2	35.72 (28.7514-45.8335)	1978 (1968-1985)
3	28.044 (26.1044-31.2183)	1986 (1983-1988)
4	23.729 (22.0482-25.9722)	1990 (1988-1992)
5	19.781 (16.8719-22.744)	1994 (1991-1997)
6	13.592 (10.3909-17.025)	2000 (1997-2004)
7	10.431 (9.1623-12.1787)	2004 (2002-2005)
8	7.741 (6.1645-9.955)	2006 (2004-2008)
9	17.215 (16.0153-19.1747)	1997 (1995-1998)
10	7.898 (5.5087-11.5448)	2006 (2003-2008)
11	3.779 (2.5841-4.9802)	2010 (2009-2011)

Multiple introductions of influenza viruses, such as A/H1N1 and A/H3N2, were reported previously [2, 19–21]. In this study, phylogenetic trees based on both the HA and NA segments and the topological structure of the Yamagata and Victoria lineages were in accordance with the conclusion that the dominant circulating influenza B viruses in the Wenzhou area from 2011 to 2014 were the result of multiple introductions from multiple locations outside of the Wenzhou area in different years or even within a single year. The HA segment in the Yamagata lineage indicated that one dominant circulating influenza B virus in the Wenzhou area during 2013 was introduced from Hangzhou, the capital of Zhejiang Province, while the other monophyletic clade in the same Yamagata lineage showed that another independent introduction in 2012 was from the Lishui area located in southwestern Zhejiang (Fig. 1). These two clades also have different times for the most recent common ancestor as estimated by the MCMC algorithm (Table 2). A similar pattern was observed for the Victoria lineage. Two introduction events related to the Wenzhou strains were from outside of Zhejiang: one was from Nanjing, Jiangsu Province, and the other was from Brisbane, Australia. Additionally, our phylogenetic analysis of the NA segment showed evidence of local persistence of several phylogenetically distinct monophyletic clades circulating in the Wenzhou area from 2011 to 2014. Viruses belonging to those clades may have been introduced from different places. For example, the strain from Brisbane shared high phylogenetic similarity to strains in Wenzhou during 2011 and 2012. There are several possible reasons for this phenomenon. Wenzhou hosts a large number of migrants, as shown in the latest government census report in 2011. They account for one third of the population in the Wenzhou area. Migrants who follow the Chinese custom of returning to their home villages during important festivals throughout the year may facilitate the transmission of viruses from one location to another. This was shown to be the case in previous studies of other viruses [2, 22–24]. Additionally, Wenzhou has connections with other regions in China and, as the third largest city in Zhejiang Province, even internationally through foreign trade, and has a higher risk of other diseases such as chikungunya. Wenzhou, and, in fact, all of Zhejiang, is a popular travel destination for both business travelers and tourists, thereby providing new channels for intercontinental introductions and transmissions. Long-term influenza surveillance is therefore essential for early detection and for providing early warning to the public of the potential for viral transmission and circulation.

This study had several limitations. First, we were unable to investigate the full epidemiological information for the samples we collected. Although we observed that children and young teenagers were the main susceptible population compared to other age groups, which was similar to findings in other studies of influenza B virus, we were unable to identify the proportion of Victoria and Yamagata in the population for each year in the Wenzhou area, which may have reflected background immunity within the population. Second, we failed to obtain the whole genome sequence for all positive samples of influenza B virus. Reassortment analysis was therefore limited. As a result, a blind area exists in the evaluation of the evolutionary processes and endemic speciation of influenza B virus in the Wenzhou area. More samples and detailed epidemiology information are needed to understand the circulation, dynamics and evolution of influenza B virus in this region.

To conclude, this study is the first to highlight the basic epidemiological and molecular characteristics of influenza B virus strains, especially the hemagglutinin and neuraminidase genes, for the 2011 to 2014 outbreak in the Wenzhou area. Amino acid substitutions were identified, and phylogenetic relationships between other strains from different locations were evaluated. Multiple introductions from outside the Wenzhou area were revealed, and potential circulation was detected. Our study revealed the variation, co-circulation and prevalence of both influenza B virus lineages during the period 2011 to 2014 in the coastal zone of southeastern China. Further studies are needed to determine the detailed dynamics and evolution of influenza B virus in this region.

## Electronic supplementary material

Below is the link to the electronic supplementary material.

Supplementary material 1 (DOC 42 kb)
