# Supplementary material for: Multiple viral introductions: molecular characterization of influenza B virus in Wenzhou, Zhejiang, China, from 2011 to 2014 based on hemagglutinin and neuraminidase genes
Source: Arch Virol. 2016 Jan 2;161:1005–13. doi: 10.1007/s00705-015-2721-7 (PMC4819752; doi:10.1007/s00705-015-2721-7)
Supplement: Supplementary file 1 — Supplementary material 1 (DOC 42 kb) [file 705_2015_2721_MOESM1_ESM.doc]

Supplementary

Table S1 Primer information for both HA and NA segment amplification

|  | Primer | segment | Sequence |
| --- | --- | --- | --- |
| 1 | 2+ | HA | GCAGAAGCAGAGCATTTTCTAATATCC |
|  | 1160 | HA | TGCCAACCTGCAATCATTCCTTCC |
| 2 | F690R | HA | AGAAGACGGAGGRCTACCAC |
|  | 1694W | HA | AGACAGATGGAGCAWGAAAC |
| 3 | F | NA | GAACAATGCTACCTTCAAC |
|  | R | NA | CAACCATTCCTCCATTACAG |

Table S2 General information for sample from Wenzhou area, Zhejiang Province, from 2011 to 2014: A, detection number, positive rate and percentage for influenza B virus; B. Age distribution for influenza B positive.

A

|  | 2011 | 2012 | 2013 | 2014 | Total |
| --- | --- | --- | --- | --- | --- |
| No. of sample | 435 | 520 | 914 | 1052 | 2921 |
| No. of Influenza B positive | 23 | 46 | 51 | 43 | 163 |
| Positive rate for Influenza B | 5.29% | 8.85% | 5.58% | 4.09% | 5.58% |

B.

| Age | 2011 | 2012 | 2013 | 2014 | Total | Percentage |
| --- | --- | --- | --- | --- | --- | --- |
| 0-5 | 4 | 10 | 2 | 9 | 25 | 15.34% |
| 6-10 | 3 | 9 | 25 | 15 | 52 | 31.90% |
| 11-15 | 1 | 1 | 15 | 1 | 18 | 11.04% |
| 16-20 | 1 | 5 | 1 | 2 | 9 | 5.52% |
| 20-60 | 11 | 20 | 6 | 12 | 49 | 30.06% |
| Above 60 | 3 | 1 | 2 | 4 | 10 | 6.13% |
| Total | 23 | 46 | 51 | 43 | 163 |  |
